# The role of recombinant human brain natriuretic peptide on the cardiac output of patients with acute decompensated heart failure using Guyton venous return curve: A STROBE-compliant retrospective study

**DOI:** 10.1097/MD.0000000000025492

**Published:** 2021-04-30

**Authors:** Jian ling Liu, Xiao fei Zhang, Zhi Liu, Jie min Li, Zhen jie Wen, Ming Zhang, Qin han Lin, Qiu ye Kou

**Affiliations:** a Department of Intensive Care Unit, Qingyuan People's Hospital,Qingyuan,China; b Department of Intensive Care Unit, The Sixth Affiliated Hospital of Sun Yat-Sen University, Guangzhou, China.

**Keywords:** acute decompensated heart failure, cardiac output, recombinant human brain natriuretic peptide, venous return curve

## Abstract

rbBNP has positive cardiac effects in patients with acute decompensated heart failure, but its effects on the systemic venous circulation are not known.

A single-center retrospective, self-controlled study was conducted on 14 patients undergone recombinant human brain natriuretic peptide (rhBNP) treatment between January 1, 2015 to December 31, 2018.

The cardiac output (CO) significantly increased from 3.75 ± 1.14 L min-1 to 4.24 ± 0.97 L min-1 30 minutes after rbBNP infusion, and to 4.20 ± 1.19 L min-1 3 hours later. The systemic vascular resistance significantly decreased from 18.85 ± 7.66 mm Hg min L-1 to 14.62 ± 6.13 mm Hg min L-1 30 minutes. The resistance to venous return (VR) significantly decreased from 5.93 ± 4.97 mm Hg min L-1 to 4.46 ± 1.53 mmHg min L-1 3 hours later. The mean systemic filling pressure significantly decreased from 32.71 ± 20.00 mm Hg to 28.254 ± 6.09 mm Hg 3 hours later.

The role of rhBNP on CO was to reduce the peripheral circulation resistance at 30 minutes after rhBNP infusion and to reduce the resistance to VR at 3 hours later.

This trial is registered at ChiCTR: ID ChiCTR1900024562.

## 1 Introduction

Cardiovascular diseases (CVDs) are major causes of the high mortality rates experienced globally. Heart failure (HF) is the terminal stage of many CVDs characterized by poor prognosis and high mortality rates. The prevalence of HF is estimated to be between 2% to 10% of the global adult population which translates to 38 million people.^[1,2]^ The costs of HF treatments in the United States were estimated to exceed US$35 billion.^[3]^ In China, the costs of HF treatments were estimated to be at $5.42 billion and the in-hospital mortality rate was estimated to be at 4.1%.^[4]^ This indicates that HF is a global public health concern.

The treatment of HF was improved through the introduction of drugs such as the angiotensin converting enzyme inhibitors, Angiotensin II Receptor Blockers (ARBs), Aldosterone antagonists and B-adrenergic receptor blocking compounds (β-blockers). The recombinant human brain natriuretic peptide (rhBNP) was approved by the FDA in 2001 to be used for the treatment of acute decompensated HF. The Brain natriuretic peptide (BNP) is a peptide hormone secreted by ventricular myocytes due to the pressure-volume overloading of ventricles.^[5]^ During the early stages of HF, there is a relative deficit of endogenous BNPs. Therefore, BNP supplements can be used to treat acute decompensated HF. The rhBNP has similar bioactive traits as BNP that influence favorable cardiac effects such as; natriuresis, diuresis, and vasodilation.^[6]^ The rhBNP also inhibits the renin–angiotensin–aldosterone system.^[7]^ Several studies reported that rhBNP has positive therapeutic effects on patients with HF however, it has some side effects such as; decreasing the cardiac preload and afterload with an increase of cardiac output (CO).^[8–10]^ The effect of rhBNP on the systemic venous circulation and its regulation on the CO have not been explored.

During the 19th century, Guyton proposed that the heart function, heart rate and stroke volume are important factors that determine CO.^[11]^ The systemic veins and venules contain 2/3 of the human body's total blood volume and this contributes to a blood volume reserve.^[12]^ The CO was largely affected by the volume of venous return (VR). Guyton and his co-workers did many experiments to show the relationship between the changes in right atrial pressure (Pra) and the changes in VR, depicted as a venous return curve.^[13,14]^ The VR is now one of the factors considered when performing hemodynamic monitoring in critical care medicine because it influences the pathophysiology of HF, shock, and vasoactive drugs. There are other factors that affect the VR such as: mean systemic filling pressure (PMSF), right atrial pressure, and the resistance to VR. The PMSF is the upstream driving pressure for VR, defined as the pressure in the vascular system when the circulatory system is at rest. However, PMSF is challenging to quantify. Mass et al 2009 presented a noninvasive way to measure of PMSF by performing 12s inspiratory-hold maneuvers in patients with mechanical ventilation at the bedside.^[15]^ These measurement procedures allowed the calculation of VR parameters from the laboratory to the real patient.

Recently, we measured the parameters of VR as a part of hemodynamic monitoring in some of those patients diagnosed with acute decompensated HF. Then some of the above-mentioned patients were treated with rhBNP. The rhBNP is effective in managing acute decompensated HF. It increases the CO and reduces the systemic vascular resistance (SVR) in the pulmonary capillaries.^[16,17]^ However, the role of rhBNP on the systemic venous circulation in patients with HF remains unclear and there are limited studies that illustrate the effects of rhBNP on CO changes using the Guyton venous return approach. Thus, a retrospective cohort analysis was performed to determine the effects of rhBNP on CO of patients with acute decompensated HF using the Guyton venous return curve approach.

## 2 Methods

This study was conducted using the clinical data of patients who were admitted since January 1, 2015 to December 31, 2018 after being approved by the Institutional Ethics Committee of Qingyuan City Hospital. A waiver for the informed consent was obtained since the data was analyzed anonymously. The inclusion criteria was as follows;

1. males/females with an age ≥18 years;
2. a history of CVDs and/or HF;
3. a diagnosis of acute decompensated HF according to the (ICD-10: I50.1);
4. patients who were in the intensive care unit requiring mechanical ventilation in the mode of volume assist–control;
5. patients who received hemodynamic monitoring by a PiCCO2 device;
6. patients who received diuretics and digoxin before being given an rhBNP intravenous infusion pump and
7. patients who had complete data of venous return curve before and after rhBNP intravenous infusion pump.

The exclusion criteria included

1. Men/women <18 years of age;
2. presence of hypovolemic, cardiogenic or vasodilatory shock, and systolic blood pressure less than 90 mm Hg;
3. presence of HF with arrhythmias;
4. presence of valvular heart disease;
5. pregnant or breast-feeding patients;
6. patients who had hepatorenal insufficiency (AST ≥80 U/L, ALT ≥80 U/L, Cr ≥127 μmol/L) and
7. patients who had incomplete information concerning their treatments.

### 2.1 Study design

This study was retrospective, self-controlled and utilized 1 center. The demographic data in this study included age, gender, heart rate, mean arterial pressure (MAP), body temperature, Acute Physiology and Chronic Health Evaluation score (APACHE II), Sequence Organ Failure Assessment Score (SOFA) and N-terminal B-type natriuretic peptide (NT-proBNP) score. All the data was derived from electronic medical records.

Patients enrolled in this study, were treated with an intravenous loading dose of rhBNP (1.5 μg kg^−1^ of body weight), followed by rhBNP infusion of 0.0075 μg·kg^−1^ min^−1^ for at least 3 hours. Each patient was given standardized preconditioning included adequate sedation and analgesia. Each patient was mechanically ventilated and had no spontaneous breathing during the measurement process. All patients were placed with a central venous catheter and a femoral artery puncture tube, which was connected with a PICCO2 monitor. The MAP, central venous pressure (CVP), CO), SVR, stroke volume (SV), and stroke volume variation (SVV) were recorded and compared at specific intervals: before rhBNP administration (T 0), 30 minutes after rhBNP administration (T 30 minutes) and 3 hours after rhBNP administration (T 3 hours). The hemodynamic parameters such as CO, SVR, SV, and SVV were obtained using the Transpulmonary Thermodilution Technique (PiCCO TM) from the Pulsion Medical Systems, (Munich, Germany). The CVP was measured by a venous catheter inserted in the right internal jugular vein by the device of Edwards PX260. The PMSF and the resistance to venous return (RVR) at each interval were calculated as described below.

**Plotting the Venous Return Curve Determining the Mean Systemic Pressure and the Resistance to Venous Return.**

The protocol for plotting the venous return curve was as follows; Firstly, all the patients was sedated by morphine and midazolam until the Ramsay Sedation Scale was at 5 or 6. The patients were mechanically ventilated in volume assist–control mode with a fraction of inspiration oxygen of 50%, respiratory rate of 16 minutes and a tidal volume of 8 mL·kg-1. Then MAP, CVP, and CO were obtained using 12s inspiratory-hold maneuvers. The above parameters were recorded at the airway plateau pressures of 5, 15, 25, and 35 cmH_2_O. The venous return curve was plotted by fitting a linear line with 4 pairs of CO and CVP. The PMSF was calculated by extrapolating the CO value to zero.^[15]^ The SVR and RVR were calculated using the following formula: SVR= (MAP–CVP)/CO; RVR = (PMSF–CVP)/CO.^[12]^

### 2.2 Statistical analysis

Statistical analysis was conducted using the SPSS 22.0 software. The continuous variables were expressed as mean ± SD or median with interquartile range. The categorical variables were expressed as frequency (n, %) and compared using the Chi-Squared test. The differences in parameters during T 0 and T 30 minutes, T 0 and T 3 hours were compared and analyzed using One-way ANOVA with repeated measures test. *P* values <.05 were considered to be statistically significant. The CO and CVP were fitted via linear regression using the least squares method as the venous return curve.

## 3 Results

### 3.1 Patients

**Figure 1:**
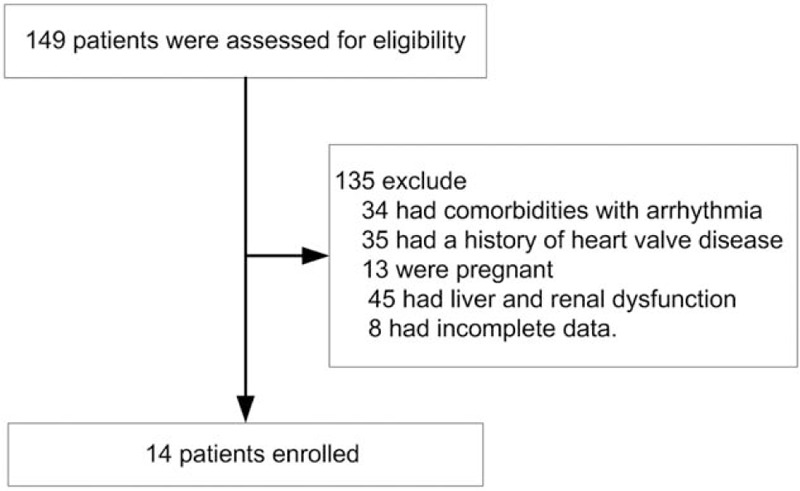
Patients’ flow-chart.

We enrolled 149 patients who had been admitted into the intensive care unit of our hospital with a diagnosis of acute left cardiac insufficiency between January 1, 2015 to December 31, 2018. A total of 135 patients were excluded, 34 patients had comorbidities with arrhythmia, 35 patients had a history of heart valve disease, 13 patients were pregnant, 45 patients had liver and renal dysfunction, and 8 patients had incomplete data (Fig. 1). This means that only 14 patients were included in this study and they had a mean age of 60.5 ± 15.3 years. The baseline characteristics of the patients were listed in Table 1. These patients had high levels of NT-ProBNP (13220.08 ± 7880.1 pg ml^−1^).

**Table 1:** Clinical characteristics and laboratory values of 14 patients.

Age (yr)	60.5 ± 15.3
Male gender (n, %)	9 (64%)
APACHE II	18.9 ± 8.7
SOFA	6.5 ± 2.2
NT-ProBNP (pg·ml^−1^)	13204.08 ± 7880.6
EF (%)	34.5 ± 3.2

### 3.2 Hemodynamic effects of rhBNP

**Figure 2:**
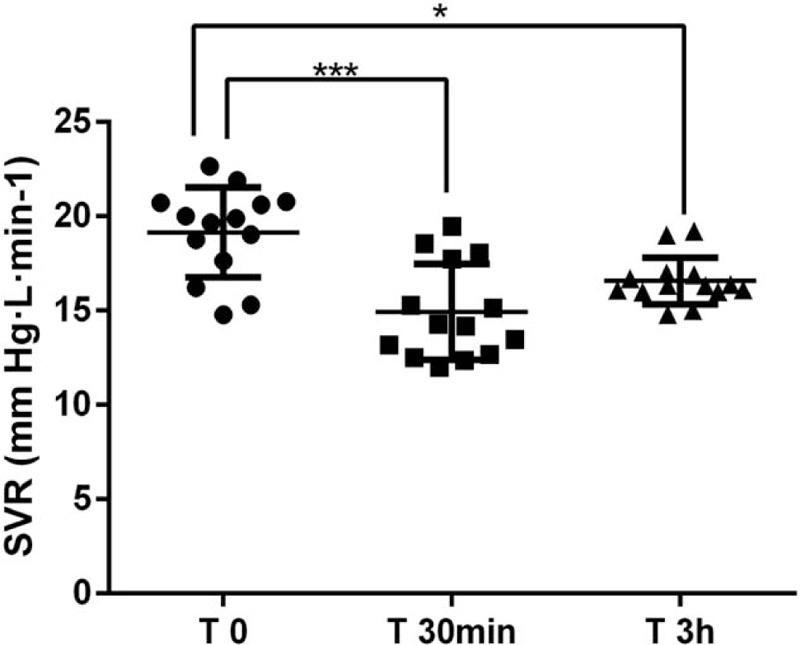
The hemodynamic parameters after 30 minutes and 3 hours of rhBNP administration. ^∗^: *P* < .05, ^∗∗∗^*P* < .0001.

**Figure 3:**
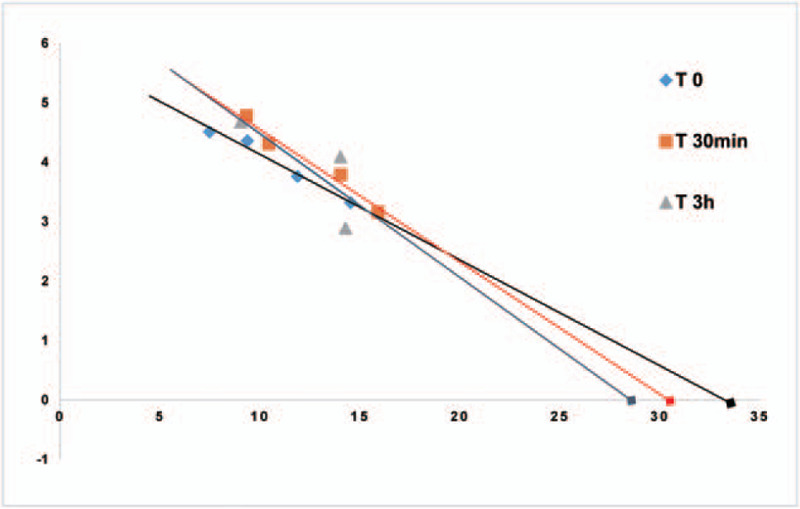
Plotting the venous return curve to calculate the values of mean systemic pressure and the resistance to venous return at bassline (T 0), 30 minutes of rhBNP administration (T 30 minutes), and 3 hours of rhBNP administration (T 3 hours).

The Table 2 and Figure 1A indicate that CO significantly increased from 3.75 ± 1.14 L min^−1^ to 4.24 ± 0.97 L min^−1^ (*P* < .05) 30 minutes after rhBNP infusion and CO evidently increased to 4.20 ± 1.19 L min^−1^ (*P* < .05) 3 hours later. The MAP showed a decreasing trend both at 30 minutes and 3 hours after the rhBNP administration, but with no statistical difference (*P* > .05) (Table 2) when compared to the baseline data (T 0). The CVP did not change values 30 minutes after the rhBNP treatment, but it significantly increased 3 hours later (*P* < .05) (Table 2). The HR showed a decreasing trend both at 30 minutes and 3 hours after rhBNP infusion, but without statistical difference(*P* > .05) (Table 2). The SV and SVV showed an increasing trend after rhBNP infusion, but without statistical difference(*P* > .05) (Table 2). The baseline values of SVR were 18.85 ± 7.66 mm Hg min·L^−1^, and it significantly decreased to 14.62 ± 6.13 mm Hg·min·L^−1^ 30 minutes after the rhBNP infusion (*P* < .05). After 3 hours it had not changed its values (Table 2 And Fig. 2D.). The PMSF significantly decreased from 32.71 ± 20.00 mm Hg to 28.254 ± 6.09 mm Hg 3 hour after infusion (*P* < .05) (Table 2 and Fig. 2B.). The PMSF showed a decreasing trend 30 minutes later, but with no statistical difference (*P* > .05) (Table 2 and Fig. 2B.). The values of PMSF were obtained from the venous return curve (Fig. 2) and they were calculated by extrapolating CO values to zero. It showed that the venous return curve shifted to left and the slope increased after rhBNP infusion (Fig. 3). The RVR had a similar trend with PMSF, it significantly decreased from 5.93 ± 4.97 mm Hg·min·L^−1^ to 4.46 ± 1.53 mm Hg·min·L^−1^ at 3 hours after the rhBNP administration (*P* < .05) (Table 2 and Fig. 2C.) but with no statistical difference after 30 minutes after treatment.

**Table 2:** The hemodynamic parameters after 30 minutes and 3 hours of rhBNP administration.

	T 0	T 30 minutes	T 3 hours
CO (L·min^−1^)	3.75 ± 1.14	4.24 ± 0.97^∗^	4.20 ± 1.19^†^
MAP (mm Hg)	75.71 ± 15.07	74.37 ± 14.81	74.25 ± 12.53
CVP (mm Hg)	11.18 ± 3.54	11.61 ± 3.44	12.30 ± 4.27
HR (min^−1^)	100 ± 25	93 ± 25	98 ± 22
SV (ml)	54.79 ± 13.44	58.80 ± 10.57	56.21 ± 1 2.11
SVR (mm Hg·min·L^−1^)	18.85 ± 7.66	14.62 ± 6.13^‡^	16.42 ± 9.04
PMSF (mm Hg)	32.71 ± 12.10	30.27 ± 11.30	28.254 ± 6.09^§^
RVR (mm Hg·min·L^−1^)	5.93 ± 4.97	5.32 ± 2.33	4.46 ± 1.53^||^
SVV (%)	9.45 ± 4.47	10.29 ± 3.92	10.18 ± 5.13

## 4 Discussion

Previous clinical studies demonstrated the favorable effects of rhBNP on the hemodynamics of patients with acute decompensated HF.^[8–10]^ The effect of rhBNP on the systemic venous circulation and its regulation on the CO had not been explored. This study was designed based on the Guyton VR theory. After an extensive literature search was done, this study is the first to explore the effects of rhBNP on acute decompensated HF using the venous return curve. The rhBNP has vasodilatory effects on the arterial and venous blood vessels, which results in reduction of preload/afterload and increased CO.^[18]^ This study showed that rhBNP improves the CO both at 30 minutes and 3-hour intervals after administration. According to the data calculated by the venous return curve, we can infer that rhBNP has effects on the arterial system at 30 minutes after administration such as: reduced SVR, cardiac afterload and increased CO. After 3 hours, the effect of rhBNP on the arterial system subsided whilst escalating on the venous system. The RVR significantly decreased, VR increased, and CO increased.

In the ancient times before Guyton, CO was proposed to be primarily dependent on the function of the heart, the heart rate and the stroke volume. The evaluation of the CO was focused on preload, afterload, contractility, and heart rate.^[19]^ The cardiovascular system is a closed loop and the heart can only pump out the blood it receives from the venous system. In the 19th century, Guyton stated that VR was an important component of CO. Based on Guyton concept, CO is dependent on the changes of the cardiac and VR functions.^[20]^ The CO = VR when the body is in a steady-state condition. According to Hagen Poiseuille law, CO equals to the difference between the mean arterial blood pressure and the central venous pressure divided by the SVR; (MAP-CVP)/SVR. The VR (Guyton analysis) equals to the difference between the PMSF and the central venous pressure divided by the resistance to VR; (PMSF-CVP)/RVR.^[12]^ From this study, there were no significant changes in PMSF, CVP or RVR values at 30 minutes after rhBNP administration when compared to the baseline values. This brings a suggestion that there was no significant increase in VR. There was a significant increase in CO at 30 minutes after administration. There were no differences in MAP and CVP as shown by (Table 2). The CO increases when there is a decrease in SVR. As shown by Table 2, there was a persistent increasing trend in CO after 3 hours of administration. Compared to the baseline values, there were no significant differences in MAP and SVR alternatively, the CVP was increased at that point. The CO declined through extrapolating the data. Constructing an averaged venous return curve for the VR allowed to discover the declining pattern of PMSF and RVR, on the contrary, CVP increased at that point. The PMSF is the upstream pressure of the VR and CVP is the downstream pressure of the VR. The difference between PMSF and CVP is the pressure gradient driving VR from the peripheral circulation to the right atrium. The reason why CO increased when the driving pressure gradient was decreasing is because the RVR decreased while VR increased.

The rhBNP is a synthetic analog of brain (B-type) natriuretic peptide and its binding to type A natriuretic peptide receptor activate guanylyl cyclase (GC). The GC subsequently induces an intracellular rise in cGMP. The cGMP activates the cGMP-dependent protein kinase (PKG) producing the vascular smooth muscle relaxation effects both on arteries and veins.^[21–23]^ This is the main reason why rhBNP exerts vasodilatory effects. From the study results, the decreased SVR and RVR attributed to the vasodilatory effects of rhBNP.

The PMSF is the upstream pressure for the VR. It is linked to the circulating blood volume, as an important component of intravascular pressure.^[24]^ The stressed volume is a decisive factor for PMSF. The stressed and unstressed volume can be converted into each other under certain conditions.^[25,26]^ In this study, the PMSF values were higher than those of other previous studies.^[27,28]^ The reason could be the fluid retention and vaso-constriction in patients with acute decompensated HF. From this study, the venous return curve shift to left and PMSF decreased after the rhBNP administration. The decrease in PMSF is the result of the stressed volume shift to the unstressed volume, due to the vasodilatory effects of rhBNP. At the same time, rhBNP has the diuretic effect, when blood volume goes down PMSF can also decrease. The rhBNP decreases the VR resistance and increases the CO increased by reducing the stress volume in acute decompensated HF.

### 4.1 Study limitations and strengths

The study had its own limitations includes the following:

1. It was a retrospective study with a small sample size.
2. The PMSF in this study was measured using the feasibility inspiratory hold method, a method that was minimally invasive monitoring at the patient's bedside and
3. the limitations of venous return curve because it reflects the steady state data rather than the dynamic indicators, which needs clinical reasoning for interpretation. The study's strength was that there was no human study that had proved no significant differences between inspiratory hold method and circulatory arresting methods except in animal models.

## 5 Conclusions

This study concluded that rhBNP improves the CO both at 30 minutes and 3 hours after administration in patients with acute decompensated HF. Through studying the the Guyton venous return curve, we could conclude that the role of rhBNP on CO was to reduce the peripheral circulation resistance at 30 minutes after rhBNP infusion and to reduce the resistance to VR at 3 hours later.

The technique of plotting Guyton venous return curve opens the door of our future studies. For example, Guyton venous return curve could be used as a tool to evaluate which kind of patients with decompensated HF could benefit from using rhBNP.

## Author contributions

**Conceptualization:** Xiaofei Zhang, Qiuye Kou.

**Data curation:** Jianling Liu.

**Investigation:** Zhi Liu.

**Methodology:** Jiemin Li.

**Project administration:** Zhenjie Wen.

**Resources:** Ming Zhang.

**Writing – original draft:** Jianling Liu, Xiaofei Zhang, Qinhan Lin.

**Writing – review & editing:** Qiuye Kou.
